# Relapsed mantle cell lymphoma manifesting with soft tissue tumors of the extremities: University of Miami experience and review of the literature

**DOI:** 10.1007/s00277-024-05997-1

**Published:** 2024-09-17

**Authors:** Iris Margalit Trutzer, Izidore S. Lossos

**Affiliations:** 1 Department of Internal Medicine, Johns Hopkins Hospital, Baltimore, MD USA; 2 Division of Hematology, Department of Medicine, University of Miami and Sylvester Comprehensive Cancer Center, 1475 NW 12th Ave (D8-4), Miami, FL 33136 USA

**Keywords:** Mantle cell lymphoma, Late relapse of lymphoma, Soft tissue lymphoma, Extranodal lymphoma

## Abstract

Mantle cell lymphoma (MCL) is frequently diagnosed at advanced stages and is characterized by multiple extranodal sites of disease, most notably the bone marrow, peripheral blood, and gastrointestinal tract. Historically the prognosis of mantle cell lymphoma has been poor with median survival of four to five years. With new treatment regimens, however, patients have been able to achieve prolonged remissions and require special attention while being evaluated for relapse. This report describes four patients treated for stage IV mantle cell lymphoma at the University of Miami who developed soft tissue relapse presenting as non-tender large masses of the extremities, including one patient who presented without associated nodal involvement. Average time to soft tissue relapse was 99 months (range: 28–240) following initial diagnosis. Providers who care for patients with mantle cell lymphoma should be aware of soft tissue lesions as a presentation of mantle cell lymphoma that merits evaluation for disease relapse.

## Introduction

Mantle cell lymphoma is a subtype of non-Hodgkin lymphoma (NHL) that accounts for approximately 5–7% of NHL diagnoses. It is characterized by a translocation between chromosomes 11 and 14, which leads to overexpression of cyclin D1 and dysregulation of the cell cycle. It is frequently diagnosed in men (70% of diagnosed cases) between ages 60–70, and median survival has historically been between 4 and 5 years. A recent study demonstrated that with the introduction of novel treatments, the 5-year overall survival (OS) improved from 68.8 to 81.6% in patients diagnosed with MCL between 2002 and 2009 compared to 2010 to 2015 [1]. MCL typically presents at advanced stage IV and involves extranodal sites such as the gastrointestinal tract, bone marrow, tonsils, and lungs [2–6].

Mantle cell lymphoma remains a challenging disease to treat and has multiple histologic subtypes with differing prognoses and disease trajectories. Classical MCL is pathologically characterized by small-to-medium sized cells with notched nuclei. Blastoid/pleomorphic MCL, diagnosed in 10–20% of MCL patients, is far more aggressive, carries a poor prognosis, and is characterized by larger cells with dispersed chromatin, high mitotic rate and common *TP53* aberrations [7]. Skin is rarely involved in MCL and is reported to be involved in only 2% of patients with blastoid MCL. Large soft tissue masses are even more rare with only 5 cases reported, primarily identified at initial diagnosis rather than at relapse [8–12]. Documented cases of patients with nodular skin disease at relapse developed recurrent disease within 3 years of initial diagnosis, and many of these patients passed away within months of diagnosis suggesting that this is a manifestation of aggressive, difficult-to-treat disease [10, 13–17]. Here we report on four patients who developed large soft tissue masses as the presenting sign of relapsed mantle cell lymphoma up to 13 years following initial diagnosis, the first series to demonstrate soft tissue lesions as a presentation of late relapse in this population.

## Case 1

**Fig. 1:**
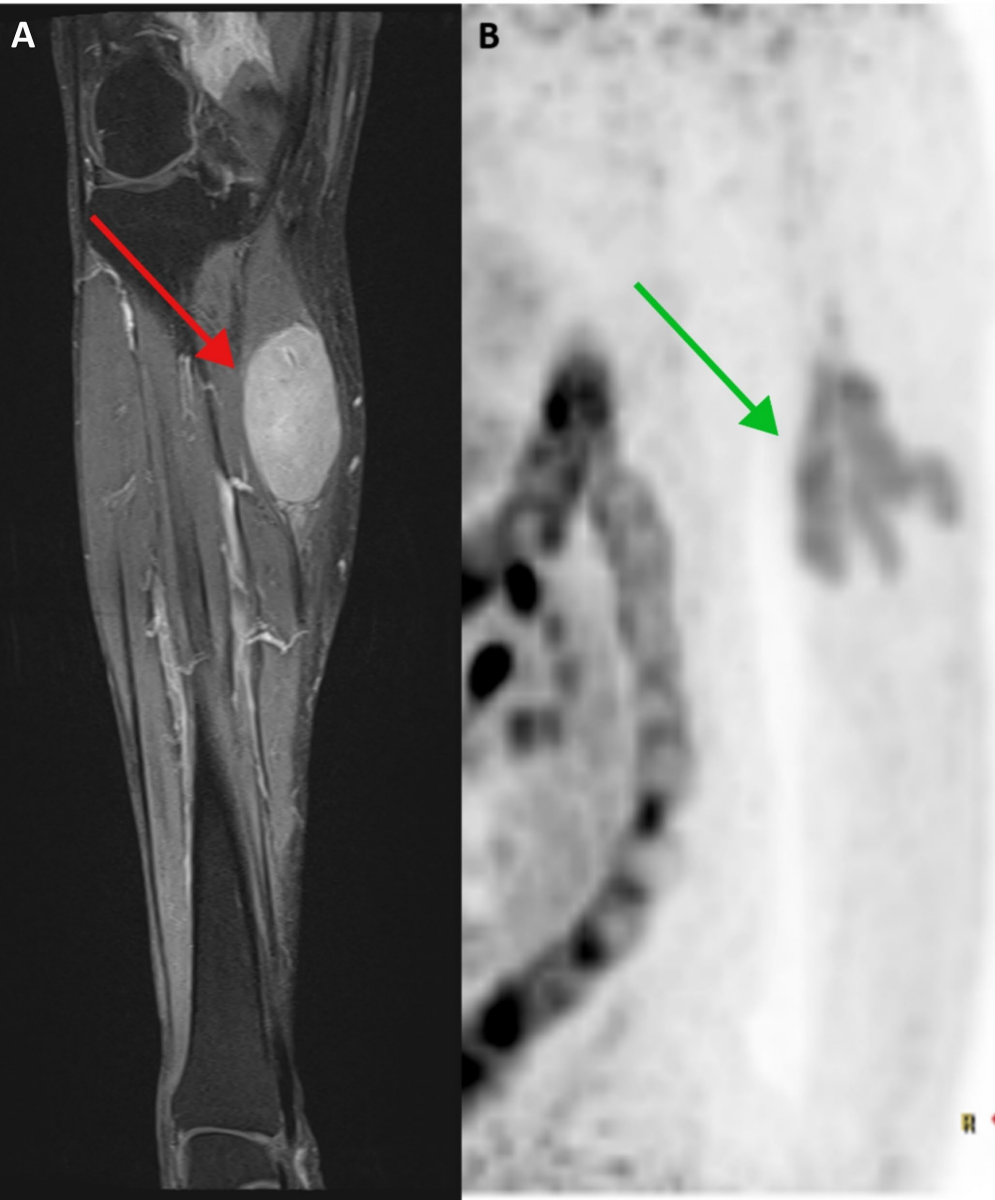
Soft tissue mantle cell lymphoma tumors. (**A**) Case 1: MRI with contrast of right tibia and fibula demonstrates oval well-demarcated T1 isointense, homogeneously enhancing mass within the substance of the proximal medial gastrocnemius muscle (red arrow); (**B**) Case 3: PET-CT demonstrating an FDG-avid soft tissue mass in the medial left elbow (green arrow)

A 56-year-old gentleman presenting in 2000 with disseminated lymphadenopathy was diagnosed with stage IV classical mantle cell lymphoma. He was treated with 6 cycles of CHOP (cyclophosphamide, doxorubicin, vincristine, and prednisone), achieving a complete remission (CR) lasting for 6-years. On relapse the patient again presented with lymphadenopathy and bone marrow involvement. Treatment with rituximab, methotrexate, doxorubicin, cyclophosphamide, vincristine, ifosfamide, cytarabine, and etoposide (R-MACLO-IVAM) was initiated, leading to CR lasting 3 years. Upon second relapse in 2009 he received bendamustine-rituximab (BR), leading to CR with a 10-year remission until relapse in 2019. He was re-treated with abbreviated BR associated with multiple infectious complications including bacterial and viral pneumonias. He successfully completed treatment and entered remission through 2023, when he developed thrombocytopenia that was diagnosed as immune mediated thrombocytopenia (ITP) at an outside hospital. Bone marrow biopsy and aspiration were negative for MCL, and the patient received multiple treatments for ITP without platelet recovery. Upon return to our clinic he was found to be thrombocytopenic (5000/ml) with disseminated mucosal and skin bleeding, inguinal lymphadenopathy, and a large asymptomatic soft tissue mass below the right knee. Due to concern for MCL relapse computed tomography (CT) scan was obtained and demonstrated lymphadenopathy of the thoracic, right pelvic, and inguinal nodes. Magnetic resonance imaging (MRI) of the tibia and fibula demonstrated a well-defined T1 mildly hyperintense, T2 isointense lesion in the medial calf, centered within the medial head of the gastrocnemius spanning approximately 7.8 cm in craniocaudal dimension and measuring approximately 3.3 × 3.5 cm (TV X AP) (Fig. 1A). There was homogeneous postcontrast enhancement of the lesion with mild perilesional enhancement within the gastrocnemius. Biopsy of the soft tissue mass demonstrated classical MCL with cells that were positive for CD20, CD5, cyclin D1 and SOX11; flow cytometry demonstrated an abnormal B cell population representing almost all B cells in the sample with surface lambda light chain restriction and abnormal expression of CD5 and CD38. Staining for p53 was negative. Before treatment for MCL was started, the patient became comatose with imaging showing large cerebral hemorrhage and died.

## Case 2

**Fig. 2:**
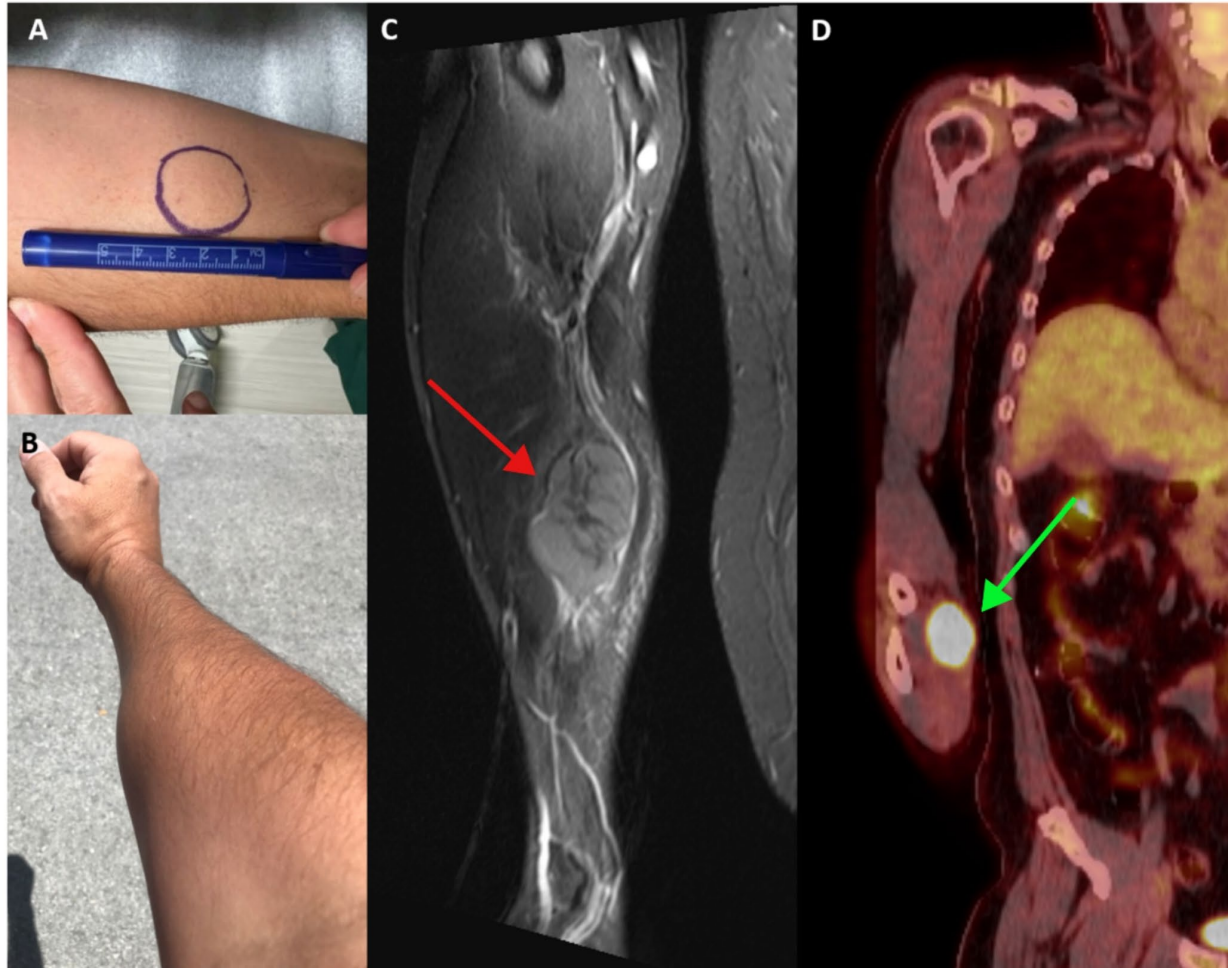
Case 2: Appearance of the soft tissue forearm lesion on the patient’s arm when it initially came to attention (**A**) and after lesion growth (**B**); **C**) MRI with contrast of right ulna and radius demonstrates intermuscular soft tissue mass within the volar and radial aspect of the mid third of the forearm, measuring 6.0 × 3.2 × 3.9 cm (CC X AP X mediolateral), with a hypo-T1/hypo-T2 non-enhancing central area (red arrow). The soft tissue mass is Fludeoxyglucose F18 (FDG)-avid on PET-CT (green arrow, **D**)

A 47-year-old gentleman initially presented with groin pain and was found to have lymphadenopathy above and below the diaphragm. An excisional biopsy of the inguinal lymph node was consistent with pleomorphic MCL. Imaging and bone marrow biopsy established stage IV disease with high MCL international prognostic index (MIPI). He was treated with 4 cycles of R-MACLO-IVAM that led to CR and was started on ibrutinib (560 mg/day) maintenance therapy. Less than 1-year post-treatment he developed a new left-shifted monocytosis and elevated lactate dehydrogenase (LDH). Bone marrow biopsy demonstrated mantle cell lymphoma with CD20, CD79a, CD5, cyclin D1, SOX11, CD10, BCL2 and p53- positive tumor cells. Salvage therapy with rituximab, dexamethasone, cytarabine, and cisplatin (R-DHAP) was started with a plan to proceed with bone marrow transplant. Following 1 cycle the patient complained about memory and word finding problems. Brain MRI showed high T1 gyriform-like lesions in the right parasagittal frontal lobe/medial frontal gyrus and cingulate gyrus, left frontal subcortical white matter, and left parasagittal parietal lobe/posterior cingulate gyrus that were associated with large areas of surrounding vasogenic edema. There were associated nodular enhancing components measuring 4x4 mm in the right frontal lobe and 8x8 mm in the left parietal lobe, respectively. Minimal linear enhancement was seen in the adjacent cingulate sulci and along the perivascular spaces in the right hemisphere. Cerebrospinal fluid analysis was negative for MCL, but brain biopsy demonstrated MCL. Following 4 cycles of high dose methotrexate with cytarabine imaging demonstrated complete resolution of brain and systemic disease. The patient proceeded with brexucabtagene autoleucel chimeric antigen receptor (CAR) -T cell therapy with an uneventful recovery. Imaging studies and repeat bone marrow biopsy on day 100 redemonstrated CR. Unfortunately, 8 months following CAR-T he presented with an asymptomatic right forearm mass that was rapidly growing (Fig. 2A and B). MRI demonstrated an intermuscular soft tissue mass within the volar and radial aspect of the mid third of the forearm, measuring 6.0 × 3.2 × 3.9 cm (CC X AP X mediolateral), with a hypo-T1/hypo-T2 non-enhancing central area measuring 2.4 × 0.8 × 2.2 cm. The mass spanned 9.4 cm in the craniocaudal dimension and approximately 4.7 cm in the transverse dimension when accounting for the peritumoral edema/enhancement (Fig. 2C). The lesion was positron emission tomography (PET) avid (Fig. 2D) without evidence of lymphoma in any other site. Biopsy of the mass demonstrated mantle cell lymphoma, blastoid variant, p53-positive with Ki67 > 90% and MYC amplification. He underwent treatment with 1 cycle of polatuzumab vedotin with good response, and was subsequently transitioned to compassionate use glofitamab completing 12 cycles and achieving CR. He is currently 8 months post-treatment and is in continuous CR.

## Case 3

A 55-year-old gentleman presented with disseminated lymphadenopathy and was diagnosed with stage IV classical mantle cell lymphoma without p53 aberrations and a high MIPI score. He was treated with 4 cycles of R-MACLO-IVAM that led to CR followed by rituximab maintenance for 3 years. Eight years following diagnosis he presented with an asymptomatic left elbow lesion and nasal congestion. Physical examination and PET-CT scan demonstrated FDG-avid lesions of the nasopharynx and soft tissue near left elbow (Fig. 1B). Biopsy of the nasopharyngeal lesion was consistent with a relapse of classical mantle cell lymphoma. He was treated with 6 cycles of BR that led to CR and was started on maintenance with ibrutinib, which he continues to tolerate after 3 years without evidence of disease relapse.

## Case 4

A 39-year-old woman presented with diffuse lymphadenopathy, B-symptoms, pleural effusion, and leukemic circulating cells. Lymph node and bone marrow biopsies as well as analysis of the leukemic cells established diagnosis of stage IVB blastic variant MCL without p53 aberrations and with high MIPI score. She was treated with R-MACLO-IVAM that led to CR and was started on thalidomide (200 mg/day) maintenance for 19 months, when she decided to stop treatment. She relapsed 34 months after initial diagnosis with an asymptomatic soft tissue mass near her left elbow that measured 3 × 4 cm. Biopsy of the lesion confirmed recurrence of blastic MCL. Staging imaging studies demonstrated disseminated disease with involvement of the chest with nodal conglomerates up to 12 cm in largest dimension and abdominopelvic involvement of the retroperitoneal, periaortic, external iliac, and left obturator lymph nodes. Bone marrow biopsy was also involved by MCL. She received 6 cycles of BR with radiation to the soft tissue mass near the elbow achieving a partial response (PR). She was evaluated for an allogeneic stem cell transplant but no suitable donor was identified. She was transitioned to rituximab, ifosfamide, carboplatin, and etoposide (RICE) with suboptimal response. She was subsequently treated with multiple regimens, including rituximab, fludarabine, cyclophosphamide, and mitoxantrone with PR, lenalidomide with PR lasting 5 months, and bortezomib without response. Then she received bendamustine, cytarabine, and rituximab that led to CR. Unfortunately, she suffered multiple infectious complications following this regimen including cytomegalovirus (CMV) infection, aspergillus, and pneumocystis pneumonias, as well as multiple bouts of gram-negative sepsis. She subsequently progressed and was treated with ibrutinib with PR until she progressed with leukemic phase and CNS disease and passed away approximately 9 years after her initial diagnosis.

## Discussion

Lymphomas in the soft tissue, either primarily within the tissues or presenting as extensions of nodal masses, have been described in the literature; these primarily arise from diffuse large B-cell lymphoma (DLBCL) and have poor prognosis [18]. This is the first case series of patients with soft tissue relapse of mantle cell lymphoma and is notable for the duration of remissions in the patients prior to the development of soft tissue relapse. These four patients represent solely 0.85% of the mantle cell lymphoma patients treated at Sylvester Cancer Center between 01/01/2005 to 01/01/2023, speaking to the rarity of this finding. Furthermore, in the 5 published reports of soft-tissue MCL, patients primarily had soft-tissue disease at index diagnosis with only two cases of relapsed disease, one with soft tissue relapse at 3 years post-index diagnosis (Table 1).

The patients in this cohort developed soft tissue relapse in the distal extremities (by the elbows, between the radius and the ulna, within the gastrocnemius muscle), consistent with published case reports that identified soft tissue lesions primarily in the extremities (Table 1). Mantle cell lymphoma involving the skin may present with small subcutaneous nodules in the proximal extremities, trunk, and face [13, 16, 17, 19–25], suggesting that location of a skin/soft lesion identified on physical exam in a patient with a history of mantle cell lymphoma cannot be used to discount the diagnosis of relapsed disease. While cases 1, 3, and 4 all had soft tissue lesions that arose alongside systemic disease, case 2 had soft tissue disease in the absence of nodal involvement (also reported in [12]). Two patients did well on therapy with a subsequent long remission (also documented in the patient described in [10]), suggesting that the presence of soft tissue disease alone is not, as skin lesions are believed to be, an indicator of difficult-to-treat disease that does not respond to standard of care therapies.

It is notable that the cases presented above did not include men in their 6th -8th decade of life, which would be consistent with the typical epidemiologic distribution of mantle cell lymphoma and literature on soft-tissue manifestations of mantle cell lymphoma. Two of the cases presented here had blastoid/pleomorphic variant, while the other 2 had classical MCL. Both patients described here with blastoid variant disease also had Ki67 expression of greater than 90%, consistent with the known aggressive nature of the disease. These patients also had relatively short remissions prior to relapse (28 and 31 months respectively), consistent with previously published reports [9]. In patients without blastoid or pleomorphic features, soft tissue disease arose 240 months (about 20 years) and 96 months (about 8 years) following initial diagnosis. In this cohort aggressiveness of subtype did not correlate with mortality as one patient with blastoid variant and one without have survived to the time of publication; CNS complications of disease were fatal in both deceased cases.

Imaging characteristics of these soft tissue lymphomas often mimic those of hemangiomas or soft tissue sarcomas; these lesions are best evaluated by MRI [26–28]. In a case series describing the MRI characteristics of soft tissue lymphomas, lymphomas were uniformly enhancing, with high signal intensity on T2 weighted imaging relative to muscle and intermediate intensity of T1 signal. They were most distinguishable from other soft tissue lesions by their depth (lymphomas were significantly more likely to be superficial lesions relative to sarcomas), were significantly more likely to have intermediate signal intensity, and were significantly more likely to have homogenous enhancement; soft tissue lymphomas did not significantly differ from other soft tissue lesions on T2 sequences [27]. In prior case reports of soft tissue lymphomas, two cases documented similar T2 hyperintense, T1 isointense tissue appearance on MRI (Table 1). In our reported patients, MRI images for one patient demonstrated a T1 isointense and T2 hyperintense lesion, consistent with the above trends, while another patient had a T1 and T2 hypointense lesion without internal enhancement. Given the heterogeneity of the imaging characteristics of soft tissue lymphomas, high index of suspicion and subsequently attention to biopsy is necessary in staging and treating patients with these lesions.

All patients in this series initially presented with stage IV disease and received aggressive therapy with R-MACLO-IVAM, a regimen which has been demonstrated to lead to long progression-free survival (PFS) and OS in patients with MCL without need for consolidation with autologous stem cell transplant (ASCT) (5-year OS of > 80%) [29]. Aggressive front-line therapy, including R-MACLO-IVAM, has been shown to improve long-term survival of patients with MCL even without consolidation and ASCT [29–34]; our data also suggests that patients treated with this regimen have enhanced responses to salvage therapy post-relapse. Past studies of patients undergoing treatment with R-MACLO-IVAM had a median progression free survival of 7.9 years and median OS not reached, with a trend towards improved survival in patients without blastoid variant disease relative to those with blastoid variant (5-year PFS in patients with blastoid variant was 20.8%, with OS of 60%). This prolonged survival, however, may lead to delayed relapses in unusual sites as reported here.

In conclusion, soft tissue masses are an infrequent but notable finding in patients with relapsed mantle cell lymphoma that merits close attention on follow up and physical examination. While some imaging findings can suggest that a soft tissue lesion is likely to represent lymphoma, close attention to biopsy is ultimately necessary to diagnose soft-tissue lymphoma. These lesions are responsive to systemic therapy and, in the patients discussed above, did not independently indicate poor prognosis. As patients are now able to attain longer remissions due to the use of up-front intensive chemotherapy, we recommend including lymphoma on the differential for enlarging soft-tissue lesions in these patients to promote early diagnosis of relapsed disease.

**Table 1:** Case reports of Mantle Cell Lymphoma within soft tissues

Source	Location of tumor	Pathology	Patient age/gender at soft tissue diagnosis	Index diagnosis or relapse	Systemic disease	Years post-index diagnosis	Tissue markers	Depth of tissue involvement	MRI Appearance	Treatment
Thorner et al., 2001 [12]	Right lower extremity	Not documented	66 / F	Index diagnosis	No	--	CD19+, CD20+, CD5+	Diffuse infiltration of tumor into bone and soft tissue	--	CHOP
Cherukuri et al., 2017 [8]	Lower back	Blastoid	61 / M	Index diagnosis	Yes	--	--	Macrolobulated soft tissue mass in the subcutis	--	R-EPOCH
Fajardo et al., 2020 [10]	Upper thigh	Not documented	73 / M	Index diagnosis	Yes	--	CD20+, cyclin-D1+, CD5+, BCl2+, Ki67 > 95%; CD3-, CD10-, CD23-, BCl6-	Superficial to rectus femoris muscle without muscle invasion	T2 hyperintense, T1 slightly hyperintense, avid, uniform enhancement	R-CHOP alternating with R-DHAOx followed by autologous SCT
Hod et al., 2019 [11]	Thigh	Not documented	65 / M	Relapse	--	Not documented	--	Medial portion of thigh, along femoral neurovascular bundle	--	Not documented
Erdem et al., 2021 [9]	Leg	Blastoid	74 / M	Relapse	Yes	3	CD20+, cyclin-D1+, SOX11+, CD5+, Ki67–80%, p53–60%	Anterior, lateral, and deep posterior compartments of the lower extremity	T2 hyperintense, T1 slightly hyperintense	Chemotherapy, not specified

CHOP: cyclophosphamide, doxorubicin, vincristine, prednisone; R-CHOP: rituximab, cyclophosphamide, doxorubicin, vincristine, prednisone; R-EPOCH: rituximab, etoposide, vincristine, cyclophosphamide, doxorubicin; R-DHAOx: rituximab, dexamethasone, cytarabine, oxaliplatin; SCT: stem cell transplant

## Data Availability

All the data is presented in the manuscript. With specific inquiries, please contact the corresponding author.
